# High-Durability Photothermal Slippery Surfaces for Droplet Manipulation Based on Ultraviolet Lithography

**DOI:** 10.3390/polym15051132

**Published:** 2023-02-24

**Authors:** Tong Wen, Chen Zhang, Yanyan Gong, Zezhi Liu, Wei Zhao, Yongjie Zhan, Ce Zhang, Kaige Wang, Jintao Bai

**Affiliations:** 1 State Key Laboratory of Photon-Technology in Western China Energy, Xi’an 710069, China; 2 International Collaborative Center on Photoelectric Technology and Nano Functional Materials, Xi’an 710069, China; 3 Key Laboratory of Optoelectronics Technology in Shaanxi Province, Xi’an 710069, China; 4 Institute of Photonics & Photon Technology, Northwest University, Xi’an 710069, China

**Keywords:** photothermal responsive, slippery surface, droplet manipulation, high durability, photo lithography

## Abstract

Photothermal slippery surface has broad applications in many research fields for noncontacting, loss-free, and flexible droplet manipulation capability. In this work, with specific morphologic parameters and modified base materials doped by Fe_3_O_4_, a high-durability photothermal slippery surface (HD-PTSS) was proposed and implemented based on ultraviolet (UV) lithography to achieve repeatability of more than 600 cycles. The instantaneous response time and transport speed of HD-PTSS were related to near-infrared ray (NIR) powers and droplet volume. Meanwhile, the durability was closely related to the morphology of HD-PTSS, which impacts the recovering of a lubricant layer. The droplet manipulation mechanism of HD-PTSS was discussed in depth, and the Marangoni effect was found to be the key factor for the durability of HD-PTSS.

## 1. Introduction

Droplet manipulating with artificial surfaces has drawn considerable attention for its important implications in plentiful fields, such as microfluidics [1,2,3], chemical synthesis [4,5], and biomedicine [6,7,8].

By controlling the wettability of the surfaces with particular structures or patterns, the droplets could be manipulated for directional transport, harvest, or storage [9,10]. For example, Ghosh demonstrated the initiative transport of droplets by a wedge-shaped superhydrophilic patterned on a superhydrophobic background [11]. However, the droplets only moved along the designed route, and the transportation was monodirectional. For a more flexible manipulation, artificial surfaces with wettability responsive to external stimuli, such as magnet [12,13,14], electrical force [15,16], and heat [17,18,19,20,21], have been proposed and developed in recent years.

Photothermal-responsive surface has been regarded as the most promising way of droplet manipulation for its high precision, noncontact, and flexibility. In 2018, a photothermal-responsive surface named paraffin-infused porous graphene film (PIPGF) was proposed and implemented for droplet transportation [22]. As NIR irradiated on the PIPGF, the paraffin would be melted to lower the sliding angle of the surface, which made the droplets slide in the route of the NIR light. Wu et al. reported a photothermal slippery lubricant-infused porous surface (SLIPS) for droplet manipulation as well. SLIPS was made from Fe_3_O_4_-doped polydimethylsiloxane (PDMS) and treated by femtosecond laser ablation to obtain micro/nanostructures for lubricant preservation. With the guidance of NIR, the droplet could be transported in an arbitrary direction and driven back and forth at least for 50 cycles [23]. Liu et al. developed a photothermal slippery tube based on PDMS and Ti_3_C_2_T_x_MXene. As the substrate heated by NIR, the wettability gradient was induced and the droplet could be repeatedly functioned for at least 20 times [24].

The results in the aforementioned works are fancy and attractive for their huge potential in numerous fields. However, in the case of PIPGF, the droplet can only be transported from a higher position to a lower position, for it was driven by gravity. On the other hand, the repeated functioning of SLIPS was quite few, and the corresponding mechanism was not clear, which might restrict its practical application.

In this work, we developed a high-durability photothermal slippery surface (HD-PTSS) for droplet manipulation based on UV lithography. Here, the durability refers to the performance of the slippery surface in repeatedly manipulating droplets. The template for HD-PTSS was fabricated by a two-step lithographing procedure and spray particle modifying. HD-PTSS can endure reciprocating transportation of a droplet more than 600 times, which is an order larger than even reported. The instantaneous response time could achieve 100 ms, and the transport speed could reach 2.8 mm/s. On HD-PTSS, droplets could be driven flexibly, even from low to high, and it was capable for the manipulation of various biological reagent droplets. HD-PTSS has prospective applications in fields such as microfluidic chips, cell culture, and chemical synthesis.

## 2. Method

A surface rich in microstructures is conducive for obtaining PTSS with high durability. Here, a composite micropillar array was designed for the slippery surface and implemented through the reverse molding of the HD-PTSS template, which was obtained by a two-step UV lithography with spray particle modifying.

### 2.1. UV Lithography

**Figure 1:**
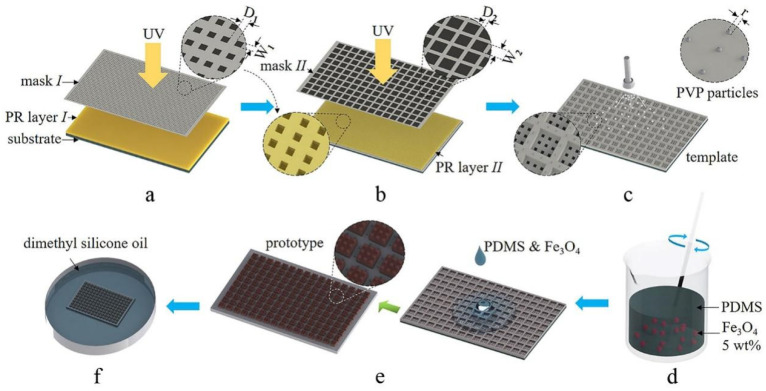
Schematic of HD-PTSS fabrication. (**a**) UV lithography step *I*. (**b**) UV lithography step *II*. (**c**) Spray particle modifying. (**d**) Photothermal colloid preparation. (**e**) Reverse molding. (**f**) Lubricant infusion.

The procedure of UV lithographing is shown in Figure 1a. In the first step, a large-scale microprotrusion array could be prepared on the top surface of HD-PTSS. The photoresist (PR) layer *I* was a spin-coated SU-8 3005 (Kayaku Advanced Materials Inc., Westborough, MA, USA) layer on silicon wafer substrates (4”) with a thickness of around 5 μm. The lithography pattern of mask *I* was a square array with a side length of 10 μm (W_1_) and an interval of 10 μm (D_1_). Since SU-8 was in negative tone, the square patterns were opaque to UV light. Then, the PR layer *I* was contact-exposed with an UV lithography system (MDA400LJ, Midas Systems Co., Ltd., Daejeon, Korea) 6 times (1 s for each exposure and 10 s as interval). The sample was baked for 10 min to sharpen the structures of the pattern after lithography. Then, the structures for microprotrusion molding were obtained after the sample was developed for 10 min with the developer and cured at 160 °C for 3 h.

The second step of the UV lithography is shown in Figure 1b, where SU-8 3035 (Kayaku Advanced Materials Inc., USA) was spin-coated as PR layer *II* on the fabrication result of the first step. Then, the patterns of mask *II* were transferred on PR layer *II* by contact-exposing 6 times (8 s for each exposure and 10 s as interval). In order to find out the key factors (e.g., height of the micropillars) to the performance of HD-PTSS, the dimension of the micropillars was adjusted with different spin-coating speeds and different mask parameters, that is, D_2_ and W_2_ in the experiment. The functioning areas were designed to 3.5 × 3.5 cm^2^ through this investigation. The sample would be post-baked for 10 min, developed for 10 min, and cured at 160 °C for 3 h after the exposure to obtain the preliminary template.

### 2.2. Template Modifying

In order to enhance the performance of the slippery surface, the preliminary template was modified by spraying polyvinylpyrrolidone (PVP) particles (K30, Sinopharm Chemical Reagent Co., Ltd., Shanghai, China) to further richen the structures of the PTSS (shown in Figure 1c). The particle solution was prepared by dispersing 10 mg PVP particles in 30 mL acetone. After the treatment of ultrasonic for 30 min, the whole solution was evenly sprayed onto the preliminary template with a spray gun. The PVP particles with diameters ranging from 10 to 20 μm would be left on the template after the acetone was volatilized at 50 °C.

### 2.3. Photothermal Colloid Preparation

Fe_3_O_4_ nanoparticle (diameter of 20 nm, Shanghai Macklin Biochemical Co., Ltd., Shanghai, China) was utilized as photothermal materials in HD-PTSS for its strong photothermal effect with NIR irradiation. Since PDMS is inherently hydrophobic, the Fe_3_O_4_ nanoparticles were processed to become hydrophobic before being doped into PDMS. First, the Fe_3_O_4_ nanoparticles were put into 3-(trimethoxysilyl) propyl methacrylate (KH570, Shanghai Yuanye Bio-Technology Co., Ltd., Shanghai, China) and stirred for 1 h. Then, the particles were extracted by centrifugation and heated at 45 °C for 6 h to remove the solvents. The processed Fe_3_O_4_ nanoparticles (5 wt%) were dispersed into PDMS prepolymer (Momentive Performance Materials Inc., Niskayuna, NY, USA) by mechanical stirring for 2 h. Finally, the photothermal colloid was obtained by putting the curing agent (10:1 ratio to the prepolymer) into the mixture and stirred for 10 min.

### 2.4. Reserve Molding and Infusion

The prepared Fe_3_O_4_-doped PDMS colloid was poured onto the template and placed in a vacuum chamber for 2 h to remove air bubbles. After the colloid was cured at 80 °C for 1 h, the solidified Fe_3_O_4_-doped PDMS film was peeled off from the template to the solidified Fe_3_O_4_-doped PDMS film from the template to obtain the prototype of HD-PTSS (see Figure 1e). Then, the prototype was infused with 1cSt dimethyl silicone oil (Dow Corning Corporation, Midland, MI, USA) for 24 h (as shown in Figure 1f). After that, it was taken out from the container and stood up on one side for 5 min to remove the extra silicone oil. The microstructures of the prototype will hold certain amount of silicone oil, and a statically balanced thin silicone oil layer will be formed on the top of HD-PTSS at last.

The droplet manipulation was observed with a contact angle meter high-speed camera (FASTCAM Mini UX100, Photron Limited, Tokyo, Japan) and CMOS camera (MER-2000-19U3M/C-L, China Daheng Group, Inc., Beijing, China). A NIR laser (HW808AD300-16GD, Shenzhen Infrared Laser Technology Co., Ltd., Shenzhen, China) with a central wavelength of 808 nm and a beam waist of 1.4 mm^2^ was adopted through the whole investigation.

## 3. Results

### 3.1. Characteristics of HD-PTSS

**Figure 2:**
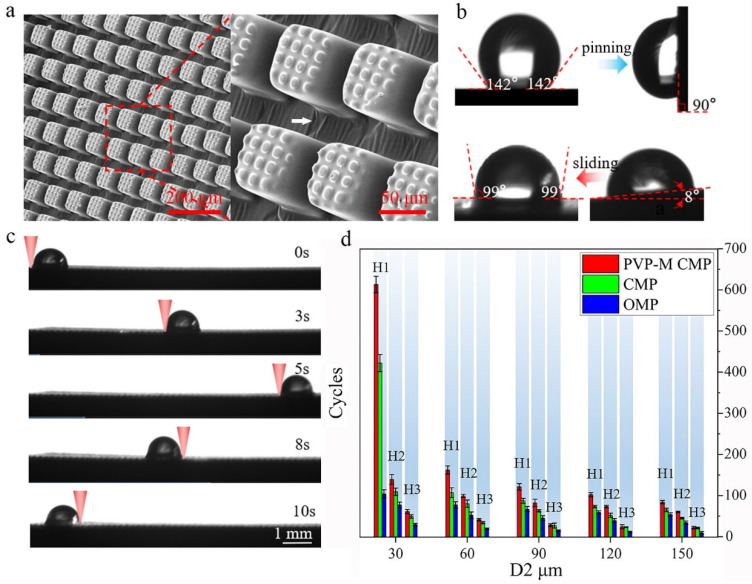
Fabrication results and performance of HD-PTSS. (**a**) Morphology of HD-PTSS. (**b**) Variation of contact and sliding angle of the functional slippery surface. (**c**) Reciprocating transportation of droplet driven by NIR light. (**d**) Droplet manipulating repetitions of HD-PTSS with different morphologic parameters.

The morphology of the surfaces was captured by scanning electron microscopy (SEM) (Apreo S, Thermo Fisher Scientific Inc., Waltham, MA, USA), as shown in Figure 2a. The desired thickness of PR layer *I* was 5 μm, and the interval (D_1_) and the width (W_1_) of the square patterns in mask *I* were all 10 μm. The thickness of PR layer *II* was 70 μm, and the interval (D_2_) and the width (W_2_) of the square patterns in mask *II* were 30 and 90 μm, respectively. It can be seen that based on the two-step UV lithography involved in this paper, a large-scale array of micropillars, which were composited with microprotrusions on the top, was successfully implemented on the slippery surface. The dimensions of the microprotrusions and pillars were consistent with the PR layer thickness and mask parameters. Further, as pointed in Figure 2a, the gully structures were formed between the composite micropillars with the treatment of spray particles, which would facilitate the preservation of the infused lubricant.

Figure 2b shows the variation of contact angle (CA) and sliding angle (SA) of the water droplet on the surface before and after silicone oil infusion. The angles were measured by a contact angle meter system (DSA100B, KRÜSS Scientific Instruments, Inc., Hamburg, Germany). The CA before the infusion of silicone oil was 142°; meanwhile, the surface had a high adhesion of water. The droplet (5 μL) could pin on the surface even the slope angle reached at 90°. Meanwhile, after being infused by silicone oil, the CA became 99°, and the SA was 8°, which was comparable to that in the reference [20]. The high-adhesive surface was switched to low-adhesive.

Figure 2c shows an example that a water droplet (5 μL) can be manipulated by NIR to go back and forth on HD-PTSS. (The video of continuous manipulation of a droplet is provided in the Supplementary Materials). When the power of the NIR light was 70 mW, the average speed of the droplet can be up to 1.48 mm/s. The laser power was much lower compared with that in the previous reports [23,24].

### 3.2. Influence of Morphologic Parameters on HD-PTSS Durability

We studied the influence of morphologic parameters, such as structural complexity, interval, and height, on the durability of HD-PTSS when manipulating water droplets. Three types of structures were adopted, which were named spraying PVP particle modified composite micropillar (PVP-M CMP), composite micropillar (CMP), and ordinary micropillar structure (OMP). Three different heights, H1 = 70 μm, H2 = 35 μm, H3 = 10 μm, were adopted for the micropillars. The height of microprotrusions on the pillars was 5 μm for simplicity. Five different intervals, which were, respectively, 30, 60, 90, 120, and 150 μm, were adopted. From Figure 2d, it is obvious that, when H = 70 μm and D2 = 30 μm, the droplet could be transported cyclically at least 614 times on HD-PTSS. In the absence of PVP particle modification (i.e., CMP structure), the score of repeated droplet manipulation was reduced to 423 times. Furthermore, in the absence of microprotrusion modification (i.e., the OMP structure), the score of droplet repeated manipulation was only 105 times. It indicates that the modifications on the micropillars are essential to the performance of HD-PTSS. This performance had far exceeded that of the so far reported photothermal responsive slippery surfaces.

### 3.3. Instantaneous Response Time and Durability with Different Laser Power

**Figure 3:**
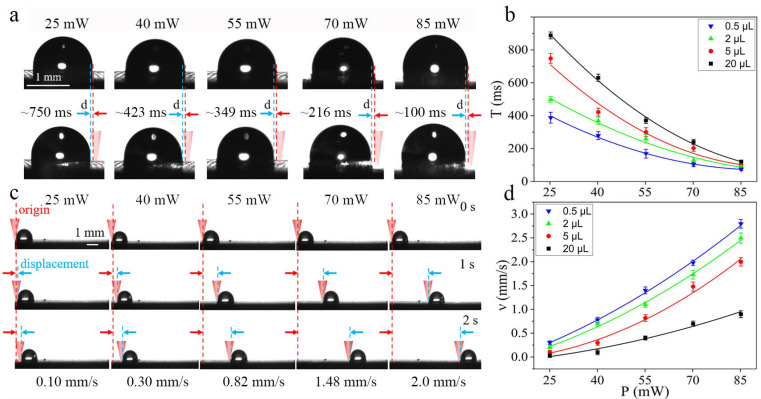
Performance of HD-PTSS irradiated with different NIR light power. (**a**) Instantaneous response time at different NIR light power. (**b**) Relationship between instantaneous response time and NIR light power of droplets with different volume. (**c**) Driving speed at different NIR light power. (**d**) Relationship between driving speed and NIR light power of droplets with different volume.

Let T be the instantaneous response time of motivating a droplet to move a perceptible distance of the naked eye, which is 100 μm, on HD-PTSS. Figure 3 shows the instantaneous response time and movement speed of the water droplet driven at different NIR light powers. As the laser power was increased, the instantaneous response times of a 5 μL droplet would be decreased from 750 to 100 ms (Figure 3a). The manipulation response is apparently enhanced by the increasing light power. This is especially important for the flexible and dynamic control of liquid droplets.

Figure 3b shows the instantaneous response time of droplets with different volumes at different laser powers. The smaller the droplet volume and the higher the laser power, the shorter the response time caused. For a water droplet of 0.5 μL, the response time could be as low as 75 ms. From the experiment, the response time (T) shows a power law relationship with the applied laser power (P) and droplet volume (V) as:

(1) $$T_{n}=-2.4*10^{4}*P^{0.01}+(V^{0.24}-V^{0.13})*(57.6-54.17*P^{0.01})+V^{0.13}+2.6*10^{4}$$

Figure 3c shows the variation of the moving speed of a 5 μL droplet at different laser powers. It is seen that the droplet would be driven faster with higher NIR light power. The moving speed can be up to 2 mm/s, which is comparable to the reports [18,23,24].

Figure 3d shows the moving speed of droplets with different volumes at different laser powers. The smaller the droplet volume and the higher the laser power, the faster the droplet transferred. With a laser power of 85 mW, the transport speed could reach 2.8 mm/s for a 0.5 μL droplet. Based on the experiment, the transport velocity (ν) shows a relationship with P and V as follows:

(2) $$\nu =(1.19*10^{-4}*P+1.57*10^{-3})*V^{2}-(2.38*10^{-3}*P+0.13)*V+0.04*P+1.64$$

**Figure 4:**
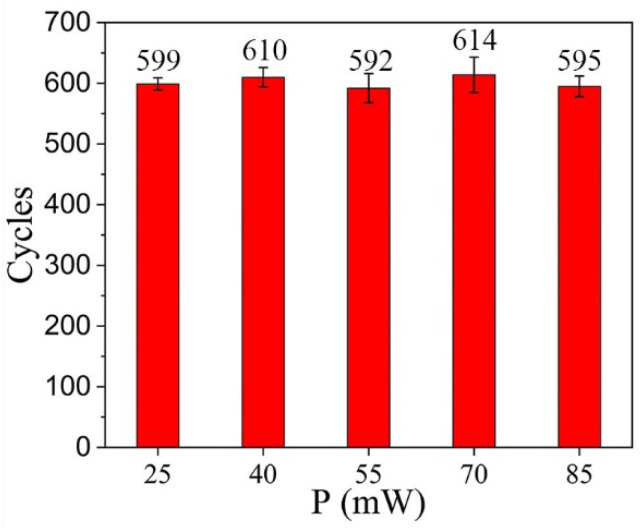
Droplet manipulation repetitions of HD-PTSS with different laser powers.

The fitting of the instantaneous response time and transport velocity is provided in the Supplementary Materials. The droplet manipulation repetitions of HD-PTSS with different laser powers are revealed in Figure 4; a water droplet of 5 μL was adopted and manipulated by the five light powers (Figure 3) on HD-PTSS. It is found that the manipulation repetitions did not change significantly while applying different powers. As shown in Figure 3d, the droplet had a higher transportation speed with high laser power compared with lower power. Since the actuating of droplets depends on a certain amount of heat, Figure 4 indicates that the heat generated by different laser powers was comparable to each other when manipulating droplets with the same volume. Therefore, the consumption of a lubricant in PTSS was similar for different laser powers. The changing of laser power had little effect on droplet manipulation repetitions.

## 4. Discussion

### 4.1. Mechanism of Droplet Photothermal Manipulation with HD-PTSS

**Figure 5:**
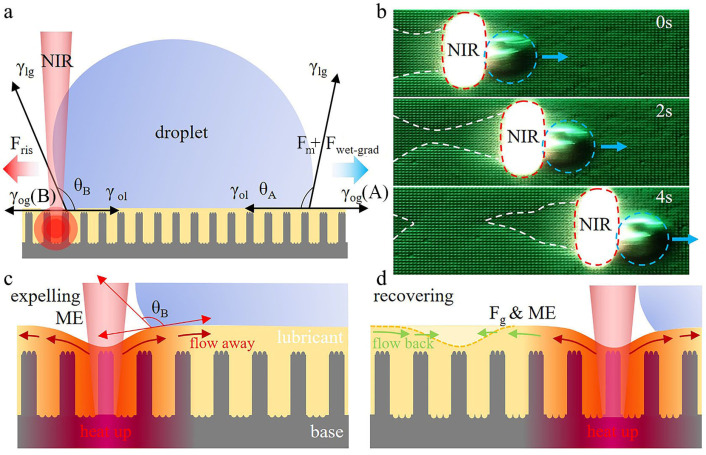
Mechanism of HD-PTSS in droplet manipulation. (**a**) Force analysis of droplet with NIR stimulus. (**b**) Expelling and recovering of silicone oil layer in droplet transportation. (**c**,**d**) Schematic of silicone oil layer motion with NIR stimulus.

The mechanism of droplet photothermal manipulation of HD-PTSS is shown in Figure 5a; suppose θ_A_ and θ_B_ are the contact angles on the two sides of the droplet. PTSS was heated up rapidly by NIR for the strong absorption of Fe_3_O_4_ nanoparticles at 808 nm. The surface tension of the lubricant would be decreased with the increasing temperature [25,26]. According to the classical Young equation [27]:

(3) $$\cos(\theta_{B})=(\gamma_{\mathrm{og}}-\gamma_{\mathrm{ol}})/\gamma_{\mathrm{lg}}$$

where γ_og_, γ_ol_, and γ_lg_ were the tensions of oil−gas, oil−liquid, and liquid−gas interfaces, respectively. The motion of the droplet on HD-PTSS is the result of the joint effect of wettability gradient force and Marangoni force. As shown in Figure 5a, when one side of the droplet was heated up by NIR, the localized oil–gas tension γ_og_ (B) was decreased, and the contact angle θ_B_ was increased simultaneously. Then, the original force balance of the droplet by the contact angles, θ_A_ and θ_B_, at the two sides of the droplet was disrupted, which initiated the wettability gradient force (F_wet-grad_) [28]. The F_wet-grad_ force always points toward the low-temperature side of a droplet. Meanwhile, due to the heat source, γ_og_ (B) on the laser-heated side was lower than γ_og_ (A) on the unheated side. This phenomena resulted in a tangential force F_m_, that is, the Marangoni force, which points forward the low temperature as well. F_wet-grad_ and F_m_ were emerged simultaneously with the changing of surface tension, and F_m_ is usually an order of magnitude smaller than F_wet-grad_ [23,29]. Suppose F_ris_ is the resistance primarily caused by the adhesion between the droplet and HD-PTSS. If it satisfies F_wet-grad_ + F_m_ > F_ris_, the droplet would be in accelerated motion. If it has F_wet-grad_ + F_m_ = F_ris_, the droplet would move with a steady velocity.

In the previous investigations, the lubricant was always regarded as a solid surface when explaining the mechanism of PTSS. However, we noticed that after the droplet was cyclically driven for a number of times, the silicone oil layer of HD-PTSS would be expelled temporarily by the laser spot and recovered in a couple of seconds, as illustrated in Figure 5b. The expelling of the silicon oil layer generally emerged after 50 cycles.

The expelling of the silicone oil layer should be primarily initiated by the Marangoni effect (ME). As illustrated in Figure 5c, when the base of HD-PTSS was heated up by NIR, the surface tension of the local silicone oil layer will be decreased; then, the silicone oil with lower surface tension would quickly flow away to the region of high surface tension because of ME. The expelling of the oil would be easily observed if the silicone oil layer surface was lower than the top of the microstructures. In addition, the actual CA in the droplet manipulation should be different from Figure 5a, for the lubricant surface was curved as the expelling of the silicone oil. On the other hand, when the droplet leaves, the silicone oil layer would flow back with the impact of ME and gravity (F_g_); then the lubricant layer could be recovered (as shown in Figure 5d).

**Figure 6:**
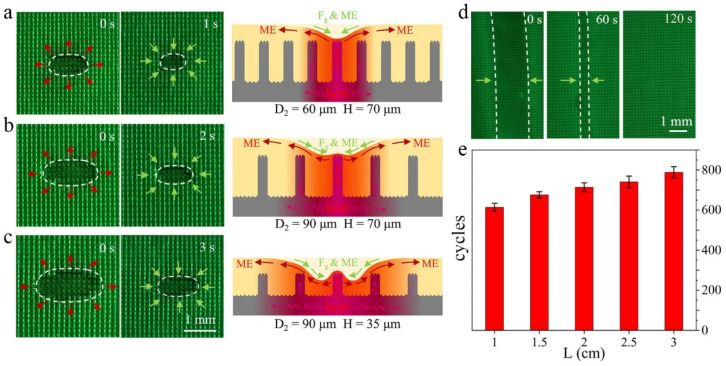
Lubricant layer recovering mechanism and its impact on HD-PTSS performance. (**a**–**c**) Expelling and recovering of silicone oil layer on PTSS with different morphologic parameters. (**d**) Recovering of silicone oil layer after droplet manipulating invalidation. (**e**) Performance of PTSS with different driving distance.

As mentioned above, we noticed that the durability of HD-PTSS should be closely related to the morphologic parameters of the surface, which influence the recovery of the silicone oil layer. The recovering of the silicone oil layer on HD-PTSS with different morphologic parameters is revealed in Figure 6a–c. The structure for the surface was PVP-M CMP, but the intervals (D2) and heights (H) of the pillars were different. It was seen that, after the three surfaces were irradiated by NIR light for 10 s, the expelling regions of the silicone oil layer were different. Among them, the surface with the parameters of H = 70 μm and D2 = 60 μm possessed the smallest expelling region and the smallest recovering time. Meanwhile, the surface with H = 35 μm and D2 = 90 μm obtained the largest expelling region and the longest time for recovering. This should be attributed to the influence of the composite micropillar structures on heat conduction. When the HD-PTSS base was heated up by NIR, the heat would be transferred to the periphery to heat up more silicone oil. Meanwhile, the photothermal zone would determine the expelling region of the lubricant layer. As shown in Figure 6a–c, if the micropillars of HD-PTSS were high and dense, more heat will be transferred to these structures; therefore, the lateral transfer distance of heat would decrease correspondingly. Otherwise, if the micropillars were low and sparse, more heat would be transferred laterally to form a larger thermal region. On the other hand, the recovering time of the silicone oil layer is positively correlated with the expelling region. The larger the expelling region, the longer the recovering time. In addition, the higher and denser microstructures would have a stronger capillary effect, which facilitates silicone oil recovering. These should be the reasons why PTSS with a higher micropillar, smaller micropillar interval, and more complex morphologic structure in Figure 2d was more durable in droplet manipulation.

While driving a droplet with HD-PTSS, the lubricant layer would become thinner and thinner for the Marangoni effect, as a droplet was moved forth and back continuously in a sole route. When the silicon oil layer was reduced to a threshold, at which F_ris_ was greater than F_wet-grad_, the droplet could no longer be driven on the surface. However, we noticed that the lubricant layer could be gradually recovered after a couple of minutes. For example, in Figure 6d, let an invalidated HD-PTSS recover for 2 min, and the oil would flow back and the cyclic driving of a droplet would recover to 50%, approximately. In addition, Figure 6e shows that after increasing the droplet driving distance to 3 cm, the surface would obtained more counts of cycles, 789 times. This is because after increasing the transportation distance, the silicone oil layer could be recovered for a longer time, which also verified the importance of recovering the silicone oil layer for the driving of the droplet.

### 4.2. Flexible Control of Droplets by HD-PTSS

**Figure 7:**
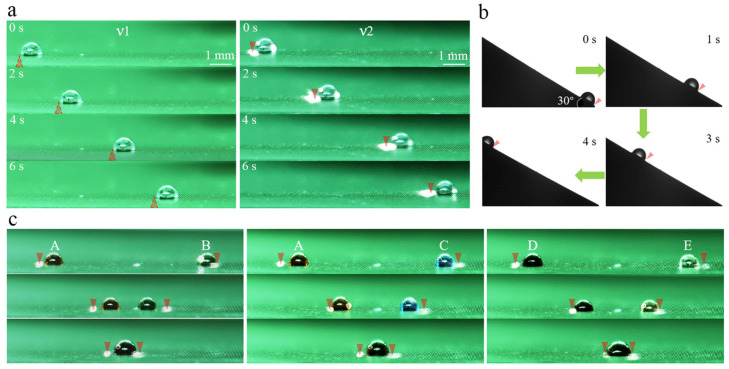
Applications of HD-PTSS. (**a**) Bottom-side manipulation for eye-safe devices. (**b**) Droplet transportation from low to high. (**c**) Biological reagent manipulation for fusion.

Figure 7 shows some promising examples of controlling the droplets on HD-PTSS with high flexibility and diversity. For example, we found that the droplet could be manipulated as NIR was projecting from the bottom side of HD-PTSS. As illustrated in Figure 7, the driving speed for a droplet of 2 μL was 1.34 mm/s (ν1) with a NIR laser of 80 mW. It was slightly slower than illuminated from the top side, which was 1.89 mm/s (ν2). However, it is still sufficiently fast for transporting droplets relative to previous reports [23,24]. Besides, the bottom-side illumination has great advantages in developing eye-safe PTSS devices and can realize on-site manipulation with commonly used inverted fluorescence microscopy systems.

As shown in Figure 7b, the droplet can be moved from the bottom to the top on an inclined HD-PTSS, with an angle of 30°. When the power of the NIR laser was 100 mW, for a droplet of 2 μL, the transportation velocity was 0.75 mm/s. It indicates that HD-PTSS could be promising for the development of fluidic devices with some complex and stereoscopic structures.

HD-PTSS was also effective for biological reagent manipulation. Figure 7c shows the manipulation of five biological samples (A–E), which are 3T3 mouse fibroblasts (A), complete medium (B), ROCK inhibitor (C), cytochalasin (D), and E-cadherin antibody (E), respectively. For clarity, the droplets were dyed with different colors. In the three groups of the fusion, all the droplets could be easily motivated to fuse with a NIR of 80 mW. The repeat manipulation counts of a biological reagent was equivalent to water droplets.

## 5. Conclusions

A high-durability slippery surface for droplet manipulation was implemented by fabricating a composite micropillar array on Fe_3_O_4_-doped photothermal material with UV lithography and spray particle modifying. As the interval and height of the pillar structures were, respectively, 30 and 70 μm, the repeated manipulation of HD-PTSS could achieve more than 600 times, which is an order larger than that in previous reports. The instantaneous response time and the transport speed of droplets were related to the laser power and droplet volume. Meanwhile, the durability of HD-PTSS was intrinsically depended by the recovering of the lubricant layer, which was closely associated with the morphologic parameters. PTSS with a more complicated morphologic structure, smaller micropillar interval, and higher micropillar would be more durable in droplet manipulation owing to a better lubricant layer recovering capacity. Furthermore, we found that the Marangoni effect was the essential factor for the durability of HD-PTSS, for it was predominant in lubricant expelling; on the other hand, it also promoted the recovering of the lubricant layer.

## Data Availability

Not applicable.

## Supplementary Materials

The following supporting information can be downloaded at: https://www.mdpi.com/article/10.3390/polym15051132/s1, Figure S1: Fitting curves of T_max_ and T_min_; Figure S2: Fitting curve of T_n_; Figure S3: Fitting curves of ν_max_ and ν_min_; Figure S4: Fitting curve of ν_n_; Video S1: Continuous manipulation of droplet.
