# Supplementary material for: High-Durability Photothermal Slippery Surfaces for Droplet Manipulation Based on Ultraviolet Lithography
Source: Polymers (Basel). 2023 Feb 24;15(5):1132. doi: 10.3390/polym15051132 (PMC10007373; doi:10.3390/polym15051132)
Supplement: Supplementary file 1 [file polymers-15-01132-s001.zip › supplementary.pdf]

polymers

MDPI

Supplementary Materials

High-durability Photo-thermal Slippery Surfaces for Droplet Manipulation based on Ultraviolet Lithography

Tong Wen [1,2,3,†], Chen Zhang [1,2,3,4,*,†], Yanyan Gong [1,2,3], Zezhi Liu [1,2,3], Wei Zhao [1,2,3], Yongjie Zhan [3,4], Ce Zhang [1,2,3], Kaige Wang [1,2,3,4] and Jintao Bai [1,2,3,4,*]

1 State Key Laboratory of Photon-Technology in Western China Energy, Xi'an 710069, China
2 International Collaborative Center on Photoelectric Technology and Nano Functional Materials, Xi'an 710069, China
3 Key Laboratory of Optoelectronics Technology in Shaanxi Province, Xi'an 710069, China
4 Institute of Photonics & Photon Technology, Northwest University, Xi'an 710069, China
* Correspondence: nwuzchen@nwu.edu.cn (C.Z.); bjt@nwu.edu.cn (J.B.)
† These authors contributed equally to this work.

Supporting Information

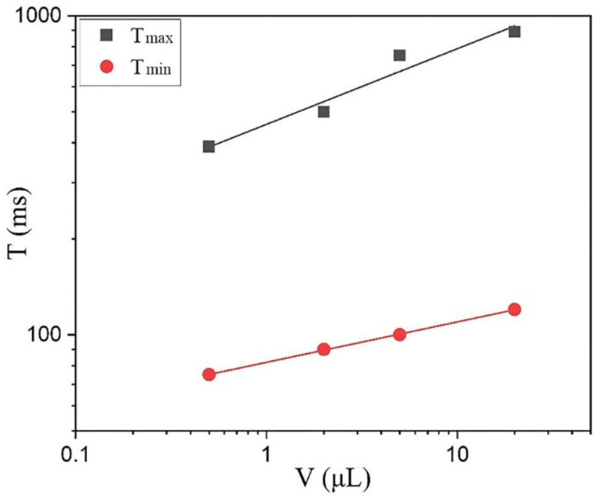

Figure S1. Fitting curves of T_{max} and T_{min}.

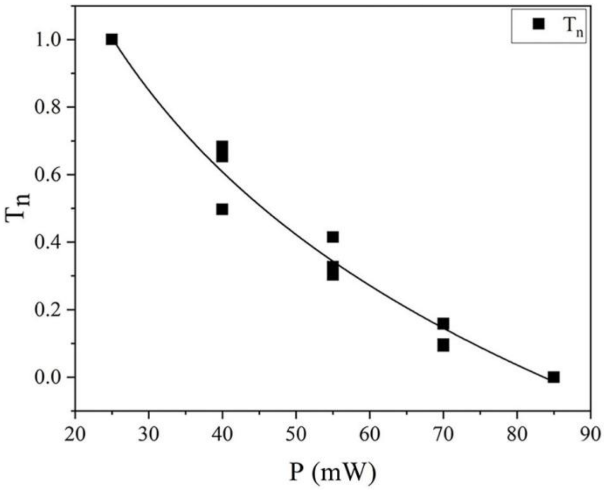

Figure S2. Fitting curve of T_n.

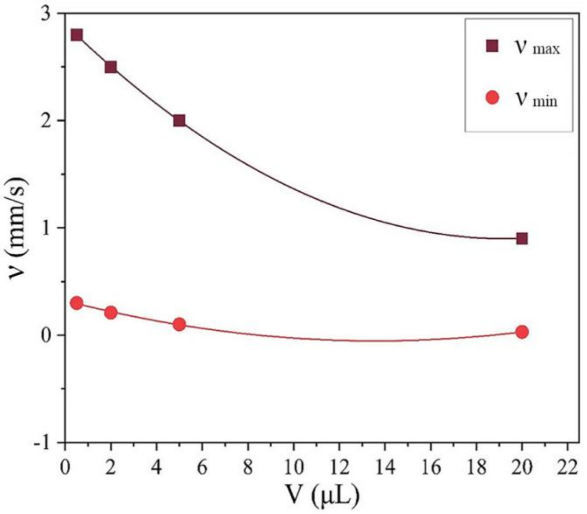

Figure S3. Fitting curves of v_{max} and v_{min}.

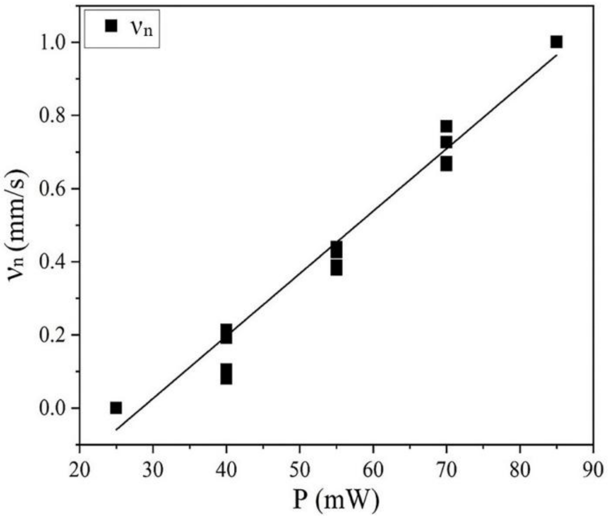

Figure S4. Fitting curve of v_n.

Letting T_{max} and T_{min} be the maximum and minimum of instantaneous response time (T) respectively. Firstly, the T_{max} and T_{min} are fitted with the droplet volume (V) in logarithmic coordinate system. The corresponding curves are revealed as Figure S1, and the expressions are as follows:

$$\log_{10}(T_{max}) = 0.24 * \log_{10}(V) + 2.66 \quad \text{(S eq. 1)}$$

$$\log_{10}(T_{min}) = 0.13 * \log_{10}(V) + 1.91 \quad \text{(S eq. 2)}$$

Then, the T is normalized to T_n and fitted with the laser power (P). The corresponding curves is revealed as Figure S2, and it has the following expressions:

$$T_n = -54.17 * P^{0.01} + 57.6 \quad \text{(S eq. 3)}$$

$$T_n = (T - T_{min}) / (T_{max} - T_{min}) \quad \text{(S eq. 4)}$$

Finally, by substituting S eq. 1 and S eq. 2 into S eq. 4, the expression of T is obtained as:

$$T_n = -2.4*10^4*P^{0.01} + (V^{0.24} - V^{0.13})*(57.6 - 54.17*P^{0.01}) + V^{0.13} + 2.6*10^4 \quad \text{(S eq. 5)}$$

On the other hand, letting ν_{max} and ν_{min} be the maximum and minimum of the transport velocity (ν). By fitting with V, the curves are obtained as Figure S3, and it has the expressions as:

$$\nu_{max} = 0.005*V^2 - 0.2*V + 2.9 \quad \text{(S eq. 6)}$$

$$\nu_{min} = 0.002*V^2 - 0.06*V + 0.32 \quad \text{(S eq. 7)}$$

Then ν is normalized to ν_n and fitted with P (as shown in Figure S4), the expression is as follows:

$$\nu_n = 0.002*P - 0.49 \quad \text{(S eq. 8)}$$

Finally, by substituting S eq. 6 and S eq. 7 into S eq. 8, the expression of S eq. 9 is obtained as:

$$\nu = (1.19*10^{-4}*P + 1.57*10^{-3})*V^2 - (2.38*10^{-3}*P + 0.13)*V + 0.04*P + 1.64 \quad \text{(S eq. 9)}$$
